# Tubeimoside 1 Acts as a Chemotherapeutic Synergist via Stimulating Macropinocytosis

**DOI:** 10.3389/fphar.2018.01044

**Published:** 2018-09-26

**Authors:** Xianling Gong, Ruibo Sun, Zhuowei Gao, Weili Han, Yawei Liu, Liang Zhao, Linlin Jing, Xueqing Yao, Xuegang Sun

**Affiliations:** ^1^The Key Laboratory of Molecular Biology, State Administration of Traditional Chinese Medicine, School of Traditional Chinese Medicine, Southern Medical University, Guangzhou, China; ^2^School of Pharmacy, Guangdong Medical University, Dongguan, China; ^3^Shunde Hospital, Southern Medical University, Foshan, China; ^4^School of Public Health, Guangzhou, China; ^5^Nanfang Hospital, Southern Medical University, Guangzhou, China; ^6^School of Basic Medical Sciences, Southern Medical University, Guangzhou, China; ^7^Traditional Chinese Medicine Integrated Hospital, Southern Medical University, Guangzhou, China

**Keywords:** tubeimoside 1, chemotherapeutic synergist, macropinocytosis, methuosis, endocytosis

## Abstract

Macropinocytosis is a highly conserved endocytic process which characterizes the engulfment of extracellular fluid and its contents into cells via large, heterogeneous vacuoles known as macropinosomes. Tubeimoside-1 (TBM1) is a low toxic triterpenoid saponin extracted from a traditional Chinese herb *Bolbostemma paniculatum (Maxim.)*. TBM1 stimulates a quick accumulation of numerous phase-lucent cytoplasmic vacuoles in multiple colorectal cancer (CRC) cell lines. These vacuoles can be termed as macropinosomes that efficiently engulf lucifer yellow. These vesicles are not overlaps with endocytic organelle tracers, such as ERTracker, LysoTracker and mitoTracker. These vacuoles induced by TBM1 partially incorporate into lysosomes. Transmission electron microscope indicates membrane ruffling to form lamellipodia. Protrusions collapse onto and then fuse back with the plasma membrane to generate these large endocytic vacuoles. Notably, TBM1 efficiently trafficks dextrans into heterotopic xenografts *in vivo*, thus provide consolidated evidence that the vacuolization can be mainly defined as macropinocytosis. TBM1 downregulates cell viability and increases PI-positive, but not highlighted Hoechst 33342-positive cells. TBM1 induced cell death can be ascribed as methuosis by hyperstimulation of macropinocytosis which can be compromised by amiloride derivative 5-(Nethyl-N-isopropyl). Light chain 3 II is recruited to these vesicles to stimulate macropinocytosis. The cell death and vacuoles can be significantly neutralized by chloroquine, but can not be the inhibited by 3-methyladenine. TBM1 can coordinate with 5-FU to exert toxicity reducing and efficacy enhancing effects *in vivo* by increasing the uptake of the latter without hepatic injury. In conclusion, TBM1 effectively induces *in vitro* and *in vivo* macropinocytosis which can traffick small molecules into CRC cells. It is an attractive drug transporter and can be harnessed as a chemotherapeutic synergist with translational potential.

## Introduction

Macromolecules are carried into the cell by invagination and pinching-off of pieces of the plasma membrane in a process termed endocytosis. Endocytosis can be largely devided into two broad categories, ‘phagocytosis’ (the uptake of large particles) and ‘pinocytosis’ (the uptake of fluid and solutes) (Conner and Schmid, 2003). Macropinocytosis is a highly conserved endocytic process by which massive membrane is ruffled and extracellular fluid is ingested concurrently (Henson, 2005). Macropinocytosis has been described as a character of Ras-transformed cancer cells. Macropinocytotic cells uptake ATP and serum album, that are abundant in the tumor microenvironment. It provides additional sources of energy and amino acids, particularly glutamine, for growth, metabolic robustness, and stress tolerance (Commisso et al., 2013; Qian et al., 2014).

Extracellular fluid and its contents are internalized into cells via large, heterogeneous vesicles known as macropinosomes (Commisso et al., 2013). So, the “fluid engulfing” habit of Ras-driven cancers can be exploited as drug delivery system for efficient uptake of anticancer agent (Reyes-Reyes et al., 2010; White, 2013). Activation of macropinocytosis facilitates the entry of Bacille Calmette-Guerin (BCG) into bladder cancer cells which dramatically increase their susceptibility to BCG infection (Redelman-Sidi et al., 2013).

Methuosis has been recently added to the panoply of cell death phenotypes. It is marked by the displacement of the cytoplasm as large fluid-filled vacuoles derived from macropinosomes (Maltese and Overmeyer, 2014). So, the loss of cell viability is preceded by extreme cytoplasmic vacuolization, caused by dysfunctional trafficking of macropinosomes. Tubeimoside 1 (TBM1) is a low toxic natural product with potent anti-tumor activity (Yu et al., 1994). Our research firstly indicates that TBM1 suppresses the viability of colorectal cancer (CRC) cells and induces the formation of macropinosomes which capturing large amount of phase-bright fluids. Thereafter, the cell death subroutine induced by tubeimoside I will be identified and the underlying mechanism of macropinocytosis will be discussed further.

## Materials and Methods

### Chemicals and Antibodies

5-N-ethyl-N-isopropylamiloride and chloroquine were obtained from Sigma-Aldrich (St. Louis, MO, United States). Tubeimoside I (National Institutes of Food and Drug Control, Beijing, China), was dissolved in DMSO (MP, France), stored at -20°C. Antibodies against Caspase-3, Cleaved caspase-3, PARP, and LC3 were purchased from Cell Signaling Technology (CST), United States. Anti-β-actin antibody was from EarthOx, LLC, San Francisco, CA, United States. Lucifer yellow, Dextran Af-488 and FITC-Dextran were obtained from Molecular Probes (Invitrogen). ER-Tracker Red, Lyso-Tracker Red and Mito-Tracker Green were purchased from Beyotime Biotechnology Corporation (Shanghai, China).

### Cell Lines and Cell Culture

Human colon carcinoma cell lines SW480 were purchased from the Chinese Academy of Sciences Committee Type Culture Collection Cell Bank (Shanghai, China). SW480 cells were cultured in RPMI 1640 medium (Gibco) supplemented with 10% fetal bovine serum (Gibco) and 1% penicillin-streptomycin (Invitrogen) at 37°C in a humidified incubator of 5% CO_2_-containing atmosphere.

### Cell Morphology and Cell Viability Assay

SW480 cells were seeded at density of 5 × 10^3^ cells per well in 96 wells plates. After treatment with or without TBM1 for different time, morphological changes were observed by phase-contrast microscopy ( Nikon Eclipse T*i*-S). To measure cell viability, MTT (3-[4,5-dimethylthiazolyl-2]-2,5-diphenyl tetrazolium bromide) (MP, France) was added into each well. Cells were incubated for 4h. The formazan crystals were dissolved in DMSO. The absorbance value was measured at 490 nm. Cell viability was normalized as the percentage of control (Wang et al., 2015).

### Western Blotting

Proteins from total cells were extracted by RIPA buffer. The whole-cell Proteins were detected by the BCA (Thermo scientific) method using the Thermo protein assay kit. Equal amount of proteins per lane were separated on 12% SDS–PAGE and transferred to PVDF membranes (Millipore). Membranes were blocked with 5% non-fat milk for 1 h at room temperature and incubated with primary antibodies overnight at 4°C. Then, incubations of HRP-conjugated appropriate secondary antibodies were performed for 1 h. Membranes were visualized by enhanced chemiluminescence (Millipore) and exposed by MiniChemi^TM^ II (Beijing, China) (Lin et al., 2014).

### Transmission Electron Microscopy

SW480 cells were treated with or without TBM1 for 12 h, After harvest, the cells were washed with cold PBS once, fixed with 2.5% glutaraldehyde at 4°C overnight, post-fixed in 1% osmium tetroxide for 2 h and subsequently dehydrated. Then samples were embedded, sectioned, doubly stained with uranyl acetate and lead citrate. Finally, Ultrathin sections were observed and captured by transmission electron microscopy (JEM1230, JEOL LTD., Japan) (Xu et al., 2015).

### Uptake of Lucifer Yellow and Organelle-Specific Tracers

SW480 cells were treated with 8 μM TBM for 24 h. The medium was removed. Cells were rinsed in HBSS (Hanks’ Balanced Salt Solution) and then incubated with 1.5 mg⋅ml^-1^ Lucifer yellow, a fuild-phase tracer, for 30 min in a 37°C, 5% CO_2_ incubator. Lucifer yellow was removed and the cells were washed once with HBSS. To label endoplasmic reticulum, mitochondria, and lysosome, respectively, ER-Tracker Red, Mito-Tracker green, and Lyso-Tracker Red were used according to the instructions supplied by the manufacturer (Beyotime, China). Phase-contrast and fluorescent images were immediately captured on an Nikon C2^+^confocal microscope.

After treatment with 8 μM TBM1 for 24 h, SW480 cells were cultured with Lyso-tracker Red for 3 h. Then, the cells were washed with phenol red - free RPMI 1640 containing 10% fetal bovine serum and incubated with 0.5 mg⋅ml-1 Dextran Af-488 in the same medium for 4 h. Finally, the cells were washed with the same medium. Phase-contrast and fluorescent images were immediately captured under confocal microscope (Nikon C2^+^).

### Time-Lapse Microscopy

SW480 cells were planted in 35 mm glass-bottom microwell culture dish. After complete adhesion, the cells were treated with 8 μM TBM1 and immediately incubated in a humidified live cell champer of 5% CO_2_-containing atmosphere at 37°C. The champer was placed on the stage of a confocal microscope (NIKON C2^+^). The phase-contrast imagines were captured every 30 s for the indicated time.

### Animal

Five to six week-old BALB/c-nu/nu male nude mice were purchased from Guangdong Medical Laboratory Animal Center and housed in Experimental Animal Center of Southern Medical university under a specific pathogen-free condition. All animal studies were performed under protocols of The Care and Use of Laboratory Animals published by the National Institutes of Health. They were approved by The Laboratory Animals Care and Use Committee of Southern Medical University. 1 × 10^7^ SW480 cells were injected into the right inguen of the mice to generate tumor xenografts. Ten days later, 50 mice were randomly divided into 5 groups. After treatment with or without TBM1, the FITC-Dextran solution was injected into tumor mass. The mice were sacrificed for the experiment, and tumors were dissected (Chunhua et al., 2013).

### HPLC Analysis of 5-FU

The tumor tissues were added with 500 μl of 0.9% NaCl and homogenated by homogenizer. An extra-centrifugation step was performed to obtain the supernatant. The supernatant was extracted using EtoAc. EtoAc extraction was dried by Nitrogen, dissolved in mobile phase and filtrated by microporous filter membrane. The assay were performed on a Shimadzu LC-20A HPLC system equipped with UV detector (Shimadzu, Tokyo, Japan). The parameter of HPLC was shown as follows. Eclinse XDB-C_18_ chromatographic column (150 mm × 4.6 mm, 5 μm) was selected. Mobile phase consisted of MeOH: H_2_O = 5 : 95 at a flow rate of 1.0 ml/min. Detection of 5-FU were performed at 260 nm.

### Statistical Methods

Parallel experiments were repeated three times. Data was analyzed using SPSS 22.0 statistical software. And results are expressed as mean ± standard deviation (SD). Mean was compared between groups using one Way - ANOVA. When the variance was homogeneous, LSD was used for multiple comparision. Dunnett T3 method was applied for the unequal variances. Mean of two groups was compared with independent samples *t*-test. A value of *P* < 0.05 indicated statistically significant difference.

## Results

### TBM1 Induces Macropinosome Formation

**FIGURE 1:**
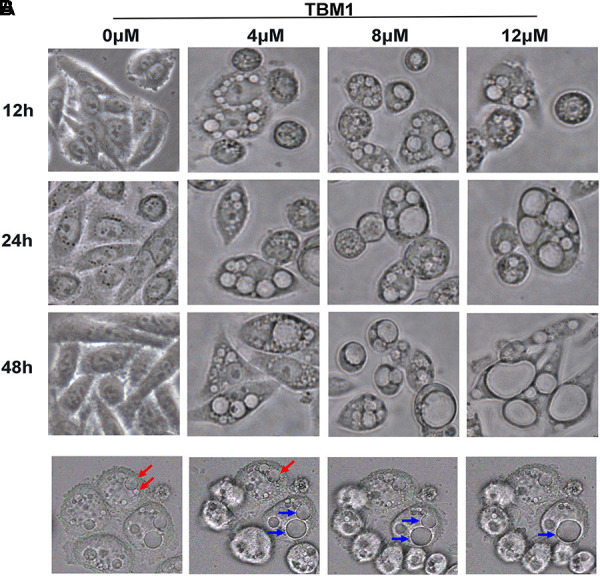
TBM1 induces accumulation of cytoplasmic vacuoles in CRC cells. **(A)** SW480 cells were treated with different concentrations of TBM1 for indicated time. Extensive accumulation of cytoplasmic vacuoles in SW480 cells were visualized using phase-contrast microscopy. **(B)** Two newly formed vacuoles fused into a larger vacuole in SW480 cells upon treatment with 8 μM TBM1. Arrowheads of the same color indicate fusion process.

To explore the underlying mechanism of TBM1 induced cell death, the morphological changes of SW480 cells were continuously monitored by phase contrast microscope. Surprisingly, a kind of phase-bright bubble-like structures that filled with liquids were formed in cells treated with TBM1. These translucent cytoplasmic vacuoles began to formed from 30 min upon TBM1 treatment (**Figure 1A**). These bubble-like vesicles were dose-dependently increased, and then gradually fused into large vacuoles with the prolongation of TBM1 treatment. The size of bubble-like structures were enlarged and the number were reduced, accordingly (**Figure 1B**).

**FIGURE 2:**
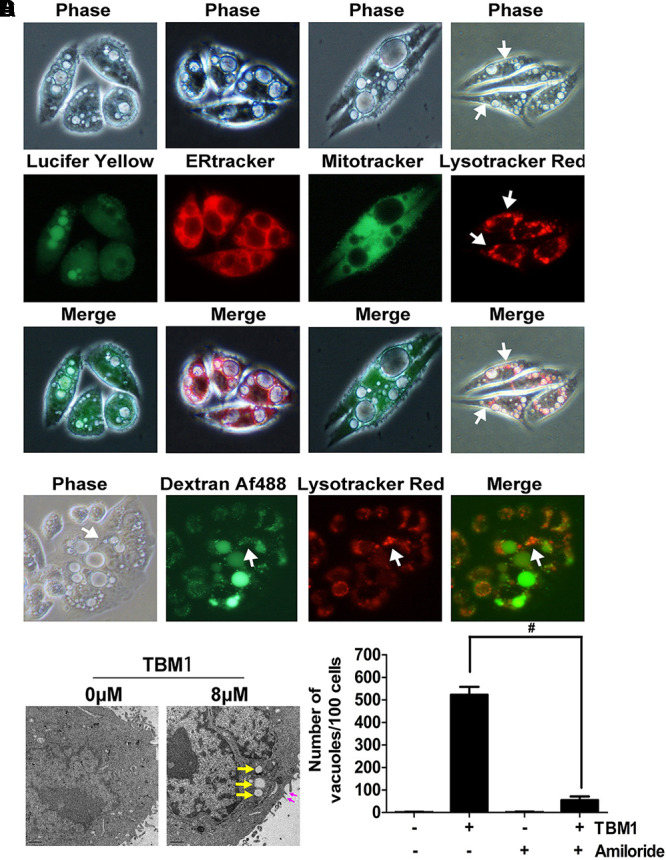
The cytoplasmic vacuoles induced by TBM1 can be identified as macropinosomes. **(A)** SW480 cells were treated with 8 μM TBM1 for 24 h and then incubated with Lucifer yellow or organelle tracers. In the left panel, some phase-lucent vacuoles incorporated Lucifer yellow. In the other panels, different organelle marker was used to label different compartment. The matching phase-contrast and fluorescent images were taken consecutively. **(B)** SW480 cells were treated with 8 μM TBM1 for 24 h and then incubated with Dextran Af-488 for 4 h, washed, and finally cultured for 1.5 h with lyso-tracker Red. The phase-contrast image exhibited the same field of cells described in the fluorescent images. **(C)** SW480 cells were treated with 8 μM TBM1 for 12 h and then observed by transmission electron microscopy. Yellow arrowheads indicated vacuoles. Purple arrowheads showed the extending lamellipodia was enclosing to form nascent macropinosomes. **(D)** SW480 cells were pre-cultured in presence or absence of amiloride (1 mM) for 1 h before exposure to TBM1 for 24 h. The number of vacuoles was counted from three different images. ^#^*p*< 0.01.

Different dyes for subcellular compartment was used to document the characters of these vacuoles. Lucifer yellow, a fluorescent dye commonly used to define macropinocytosis, incorporated into the bubbles upon TBM1 treatment (**Figure 2A**, left panel), suggested that TBM1 induced fluid phase uptake might be macropinocytosis. Further, no substantial overlap was observed between the phase-lucent vacuoles and the compartments labeled with ERTracker Red, a tracer of endoplasmic reticulum. Similar results were obtained when the cells were stained with MitoTracker Green (MTG), a mitochondrial-selective fluorescent label (**Figure 2A**, central panel). However, a small portion of overlap could be found between the phase-lucent vacuoles and the lysosome compartments labeled with LysoTracker Red (**Figure 2A**, right panel). The coupling between TBM1 induced vacuoles and lysosome was further evidenced by the combined use another fluid-phase marker dextran 488 and lysotracker (**Figure 2B**). The above observations were repeatable in DLD-1 cells, either (**Supplementary Figure S1**). These results were consistent with previous report that the overlay between macropinosomes and lysosomes that traffick fluid-phase cargo to the latter (Dolat and Spiliotis, 2016).

Lamellipodia was found closing around regions of extracellular fluid to form nascent macropinosomes in cells treated with TBM1 by electron micrographs. The numerous large cytoplasmic vacuoles were morphologically similar to the macropinosomes (**Figure 2C**). Notably, amiloride derivative 5-(Nethyl-N-isopropyl) (EIPA), a selective inhibitor of macropinocytosis, significantly reduced the vesicles induced by TMB1 (**Figure 2D**). Taken together, these results support the conclusion that the majority of the phase-lucent vacuoles can be defined as macropinosomes, and some of them fused with lysosomes.

### TBM Trafficks Dextrans Into Heterotopic Xenografts *in vivo*

**FIGURE 3:**
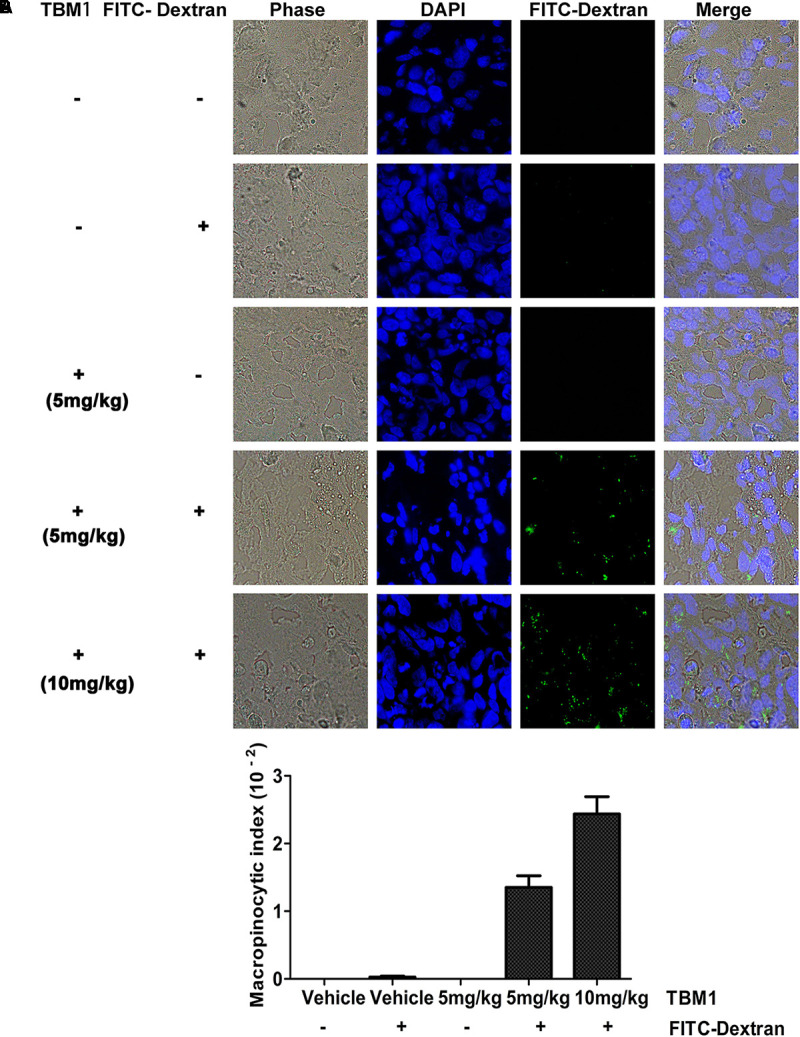
TBM1 induces engulfment of extracellular fluid into tumor tissue *in vivo* with FITC-Dextran labeling. **(A)** Tumors from five groups were dissected, fixed, embedded and sectioned. Nuclei are stained with DAPI. Fluorescent images exhibited that FITC-dextran was incorporated into cytoplasmic vacuoles *in vivo* in mice treated induced-TBM1. Photos were taken by fluorescence microscope (400 ×). **(B)** Macropinocytic index was calculated as: total particle area / total cell area. Total particle area and total cell area were measured by ImageJ software.

Macropinocytosis can be harnessed as a drug-delivery strategy to uptake both solid cargo and fluid. To determine the macropinocytic efficiency in xenograft tumors *in vivo*, SW480 cells were injected subcutaneously into immunodeficient mice as described previously (Commisso et al., 2014). Fluorescein isothiocyanate (FITC) conjugated dextrans internalized into the tumor efficiently in the presence of TBM1 (**Figure 3A**). Nearly no trafficking events was observed in mice treated with TBM1 or FITC conjugated dextrans alone (**Figure 3B**). These results suggested that TBM1 is efficient to internalize lower molecular weight cargo into mice tissues *in vivo*.

### TBM1 Inducing Necrosis in CRC Cell Lines

**FIGURE 4:**
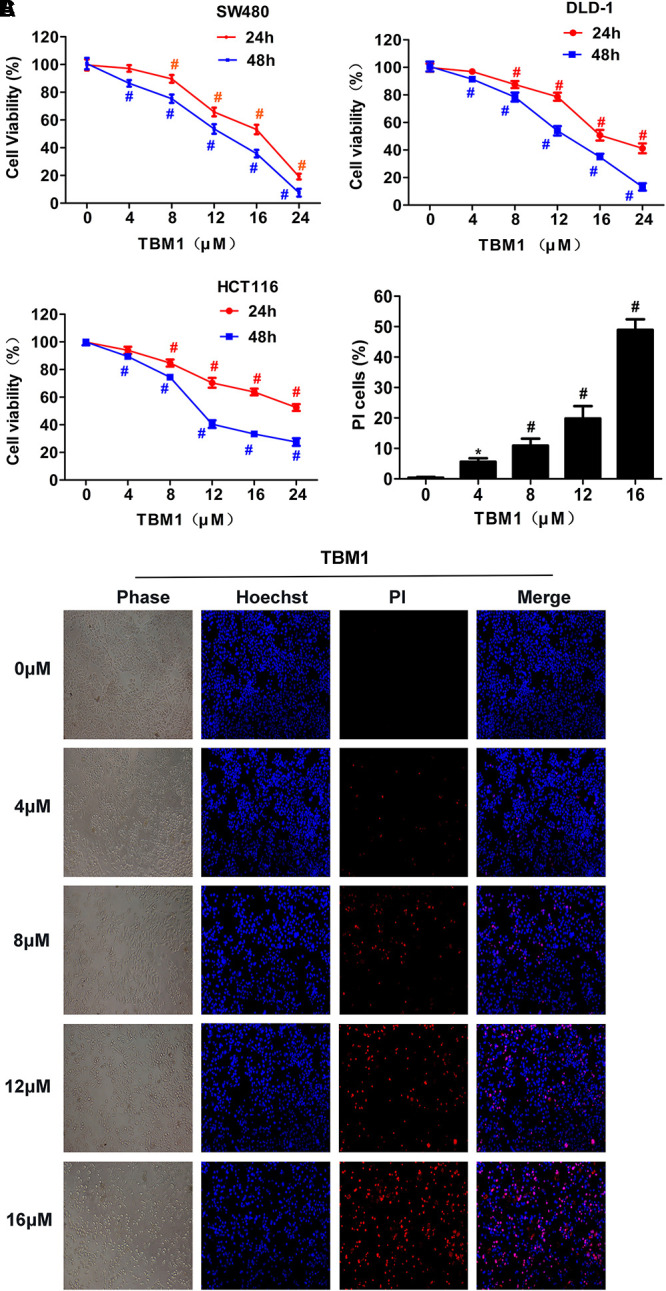
TBM1 triggers necroptotic cell death in multiple CRC cells. **(A)** The CRC cell lines were treated with different concentrations of TBM1 for 24 or 48 h. Cell viability was measured by MTT assay (*n* = 5). **(B)** After treatment with TBM1 for 48 h, SW480 cells were stained Hoechst and PI. PI positive cells were analyzed fluorescence microscope. **(C)** Fluorescent images were captured by fluorescence microscope (100×). Data presented were showed as means ± SD. ^∗^*p*< 0.05, ^#^*p*< 0.01.

To further evaluate the effects of micropinocytosis on cell death, cell viability was assayed in human colon adenocarcinoma cell lines SW480, DLD-1 and HCT116. The viability of all three cell lines was significantly reduced by TBM1 in dose- and time-dependent manner (**Figure 4A**). SW480 cells were then double stained with cell-membrane permeable DNA binding dye Hoechst 33342 and cell-membrane impermeable propidium iodide (PI) (**Figure 4B**). Interestingly, PI-positive, but not highlighted Hoechst 33342 positive, SW480 cells were significantly upregulated with the dose escalation of TBM1 (**Figure 4C**). These results suggest that the lowered cell viability might be caused by the upregulation of cell necrosis in the condition of TMB1 treatment.

### TBM1 Hyperstimulates Macropinocytosis to Induce Methuosis

**FIGURE 5:**
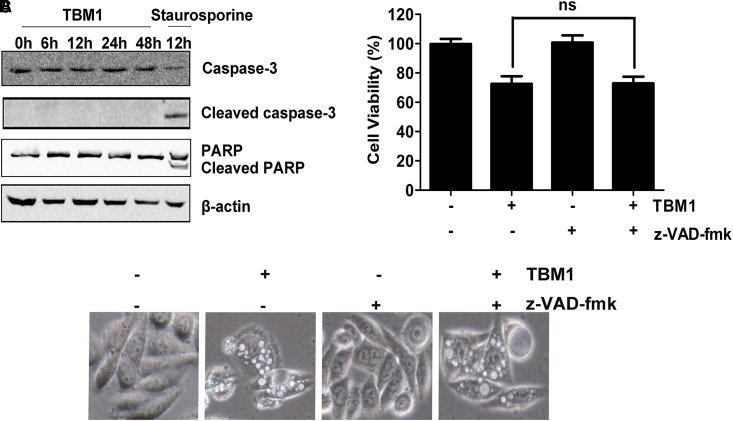
TBM1 induces Caspase-independent cell death in CRC cells. **(A)** SW480 cells were treated with 8 μM TBM1 for different time or 1 μM staurosporine for 12 h. Protein expression of caspase 3, cleaved caspase 3, PARP and cleaved PARP was detected by Western blotting. Beta-actin were served as loading control. **(B)** SW480 cells were pre-cultured with or without pan-caspase inhibitor, z-VAD-fmk (50 μM) for 1 h before exposure to TBM1 for 48 h. Morphological changes were observed by phase-contrast microscopy. **(C)** Cell viability was measured by MTT assay (*n* = 5). Data presented were showed as means ± SD. ns means that there are no statistics differences.

To clarify the subroutine of TBM1 induced cell death, SW480 cells were pre-incubated with 50 μM zVAD-fmk (zVAD, a pan-caspase inhibitor) for 1 hr followed by treatment with TBM1. As showed in **Figure 5A**, cleavage of caspase 3 and PARP were highly stimulated by staurosporine, a pro-apoptotic agent, but can not be activated by TBM1. The vacuolization (**Figure 5B**) and cell death (**Figure 5C**) induced by TBM1 can not be compromised by zVAD pretreatment, suggested that TBM1 induced cell death is not apoptosis. The cell death induced by TBM1 upon zVAD pretreatment was repeated in DLD-1 cells (**Supplementary Figure S2**).

The quick and striking accumulation of numerous phase-lucent cytoplasmic vacuoles might lead to cell death (Maltese and Overmeyer, 2015). Notably, it also compromised the cell death inducd by TBM1 accompanied by the suppression of vacuolation structures. Morphological observation showed that TBM1 increased remnants of ruptured cells. The nuclei were mainly intact and contained diffuse chromatin and a prominent non-fragmented nucleolus. These observations indicated that tubeimoside I induced cell death resemble the necrosis-like forms rather than classic apoptosis. Together, TBM1 stimulates macropinocytosis which extreme expansion of macropinosomes might lead to methuosis (Park et al., 2016).

### TBM Recruits LC3-II to Induce Macropinocytosis

**FIGURE 6:**
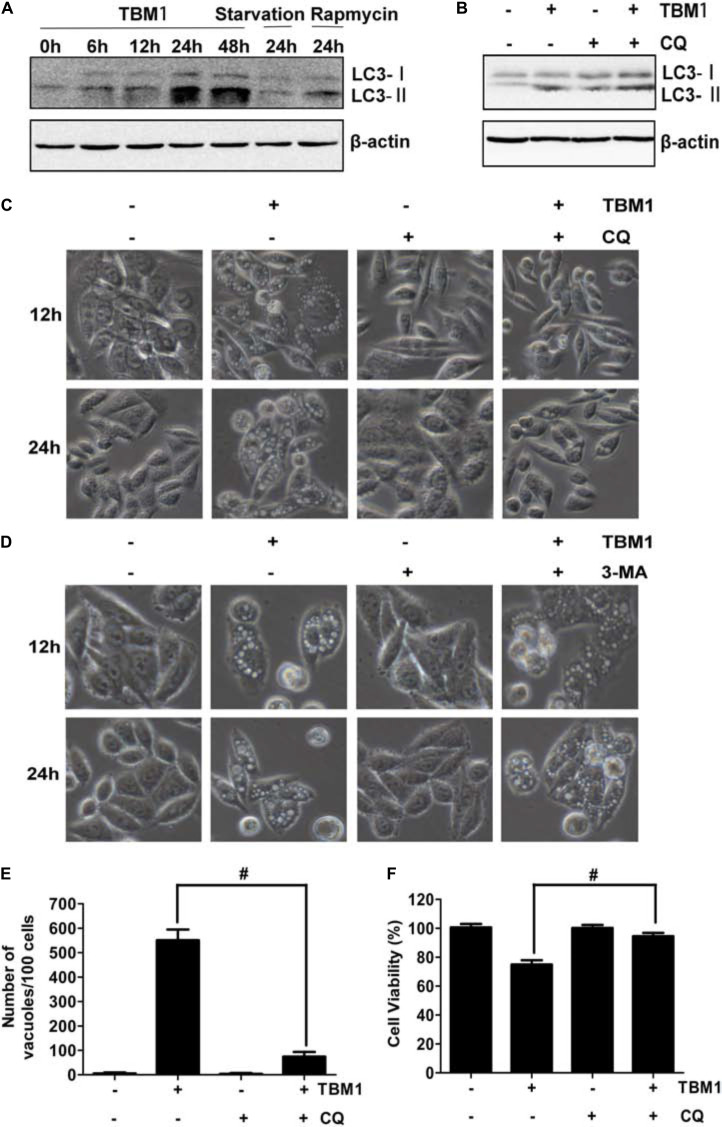
Chloroquine suppress TBM1-induced vacuolation and cell death in CRC cells. **(A)** SW480 were treated with 8 μM TBM1 for different time or starvation or 1 μM rapamycin for 24 h. Protein expression of LC3-I and LC3- II was detected by Western blotting. Beta-actin were served as loading control. **(B)** SW480 cells were pre-cultured with or without chloroquine (CQ) (10 μM) for 1 h before exposure to TBM1 for 24 h. LC3 processing was assessed using Western blotting analysis. **(C)** After treatment with SW480 cells for different time as (B), morphological changes were observed by phase-contrast microscopy. **(D)** SW480 cells were pre-cultured with or without 3-MA (3 mM) for 1 h before exposure to TBM1 for different time. Morphological changes were observed using phase-contrast microscopy. **(E)** The number of vacuoles of the same cultures as (B) for 48 h was counted from three different imagines. **(F)** Cell viability was measured by MTT assay (*n* = 5). ^#^ *p*< 0.01.

It has been noted that macropinosomes are targeted by light chain 3 (LC3) in a manner dependent on autophagy proteins, but independent of autophagosomes (Florey et al., 2011). Our results showed that the amount of membrane-bound LC3-II (the phosphatidylethanolamine-conjugated form) was significantly upregulated by TBM1 (**Figure 6A** and **Supplementary Figure S3**). It was also elevated by serum starvation and rapamycin, two classic autophagy inducers (Park et al., 2016). The expression of LC3-II was enhanced by chloroquine (CQ) (**Figure 6B**). Consistent with the previous report, TBM1 induced vacuoles can be significantly reduced by CQ (**Figures 6C,E** and **Supplementary Figure S3**), but can not be the inhibited by autophagosome inhibitor 3-MA (**Figure 6D**). The lowered viability induced by TBM1 can also be compromised by CQ pretreatment, either (**Figure 6F**). Taken together, LC3 is recruited in the hyperstimulation of macropinocytosis which leads to declined viability (Florey et al., 2011).

### TBM1 Increases 5-FU Intake to Exert Synergistic Anticancer Effects

**FIGURE 7:**
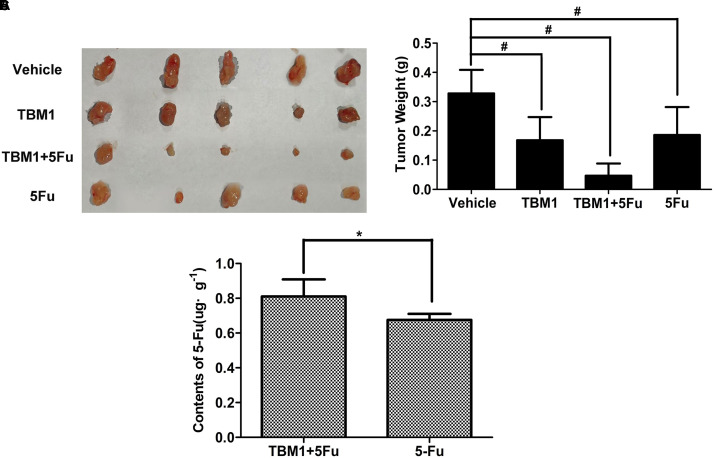
TBM1 coordinates with 5-FU and enhances the chemotherapy effect of 5-FU in mice with CRC xenografts. **(A)** The illustration of tumors from four groups. **(B)** Tumor weight was measured and analyzed. **(C)** The contents of 5-Fu in cancer tissue of mice by TMB1 or the combination of TBM1 and 5-Fu after 1 h. ^#^*p*< 0.01, ^∗^*p*< 0.05.

To explore the potential anti-neoplastic of TBM1, the direct and synergistic antitumoral effects of TBM1 was further tested in tumor-bearing mice *in vivo*. The tumor size was only mildly lowered by TBM1 or 5-FU treatment alone. However, the tumor growth was significantly suppressed by 5-FU and TBM1 treatment synergistically (**Figure 7**). Together, TBM1 might coordinates with anticancer agents to amplify their anticancer efficacy.

We hypothesized that TBM1 enhances the anticancer effects of 5-FU by increasing its internalization. HPLC analysis showed the contents of 5-FU were significantly promoted in tumor tissues pretreated with TMB1 (**Figure 7C** and **Supplementary Figure S4**). Furthermore, the liver function tests showed that the level of serum alanine amino-transferase (ALT), aspartate amino-transferase (AST), alkaline phosphatase acid (**Supplementary Table S1**) are all in the normal range value. Taken together, TBM1 synergizes the anticancer effects of 5-FU by increasing the internalization of the latter without hepatic injury.

## Discussion

Tubeimoside induces the formation of numerous vesicular-like structures rapidly. The large electron-lucent vacuoles were clearly distinct from smaller structures fitting the description of classic autophagosomes, which have double membranes surrounding luminal cytoplasmic contents (Seo et al., 2016). By labeling endocytic organelles with ERTracker, LysoTracker and mitoTracker, endosomes and mitophagy can be ruled out (Valdivia et al., 2017). Our results are consistent with the characterization of a minimal fusion between macropinocytotic vacuoles and lysosomes (Overmeyer et al., 2008). The examination with transmission electron microscope indicates protrusions collapse onto and fuse back with the plasma membrane to generate large endocytic vesicles (Marques et al., 2017; Valdivia et al., 2017). The vacuoles were generally devoid of cytoplasmic components or organelles, although some contained unidentified membranous inclusions or small quantities of amorphous electron-dense material. Uptake of lucifer yellow provides consolidated evidence that TBM1 induced vacuolization can be mainly defined as macropincytosis which subsequently leads to methuosis (Manara et al., 2016; Cingolani et al., 2017).

Morphological analysis indicates that nuclear fragmentation, reduction of cellular and nuclear volume, and plasma membrane blebbing can not be observed in cells treated with TBM1. zVAD pretreatment can not rescue the cell death, suggest that TBM1 induced cell death is distinct from apoptosis (Kroemer et al., 2009; Galluzzi et al., 2011). Cytoplasmic swelling and moderate chromatin condensation indicate that TBM1 induced cell death resembles the character of necrosis (Kroemer et al., 2009). Necrosis has long been accepted as a passive or accidental cytolytic process provoked by cell injury or metabolic catastrophe. However, it has been updated recently that necrosis can be regulated by some specific inhibitors (Degterev et al., 2005; Galluzzi and Kroemer, 2008; Cho et al., 2009). So, although still controversial, TBM induced methuosis represents a novel cell death and might be a subtype of necrosis (Cai et al., 2013; Maltese and Overmeyer, 2014; Diederich and Cerella, 2016).

Tubeimoside induces the formation of single-membrane vacuoles that are different from double-membrane autophagosomes. However, LC3 recruitment is indispensable for the formation of macropinocytic vacuoles (Florey et al., 2011). Further evidence show that macropinosomes are targeted by LC3, in a manner dependent on autophagy proteins, but independent of autophagosomes. Chloroquine increases the autophagosomes as it inhibits their fusion with lysosome. However, it suppresses number of macropinosomes, suggested that TBM1 induced vacuoles are different from autogosomes (Seo et al., 2016).

Our research provides consolidated evidence that TBM1 induces macropinocytosis *in vivo* and *in vitro.* Notably, it coordinates with 5-FU and enhances the chemotherapy effect of the latter. So, TBM1 can be harnessed as a chemotherapeutic synergist by inducing vacuolization. It is also an attractive natural molecular probe to explore the potential link between macropinocytosis and methuosis (Sun et al., 2017). In conclusion, TBM1 is a natural drug transporter that functions as chemotherapeutic synergist via stimulating macropinocytosis.

## Author Contributions

XG and XS designed the experiments, supervised, and participated in the entire work. XG, RS, ZG, YL, LZ, and LJ maintained and performed cell and animal studies, microscopic observation, and Western blot. WH supported fluorescence analysis. YL, LZ, and XY contributed to data analysis. XS wrote and edited the manuscript. RS and XS revised the manuscript. All the authors read and approved the final manuscript.

## Conflict of Interest Statement

The authors declare that the research was conducted in the absence of any commercial or financial relationships that could be construed as a potential conflict of interest.
