# Corrigendum: Tubeimoside 1 Acts as a Chemotherapeutic Synergist via Stimulating Macropinocytosis

**DOI:** 10.3389/fphar.2021.730775

**Published:** 2021-07-12

**Authors:** Xianling Gong, Ruibo Sun, Zhuowei Gao, Weili Han, Yawei Liu, Liang Zhao, Linlin Jing, Xueqing Yao, Xuegang Sun

**Affiliations:** ^1^ The Key Laboratory of Molecular Biology, State Administration of Traditional Chinese Medicine, School of Traditional Chinese Medicine, Southern Medical University, Guangzhou, China; ^2^ School of Pharmacy, Guangdong Medical University, Dongguan, China; ^3^ Shunde Hospital, Southern Medical University, Foshan, China; ^4^ School of Public Health, Guangzhou, China; ^5^ Nanfang Hospital, Southern Medical University, Guangzhou, China; ^6^ School of Basic Medical Sciences, Southern Medical University, Guangzhou, China; ^7^ Traditional Chinese Medicine Integrated Hospital, Southern Medical University, Guangzhou, China

**Keywords:** tubeimoside 1, chemotherapeutic synergist, macropinocytosis, methuosis, endocytosis

In the original article, there was a mistake in Figure 6 as published.

**FIGURE 6:**
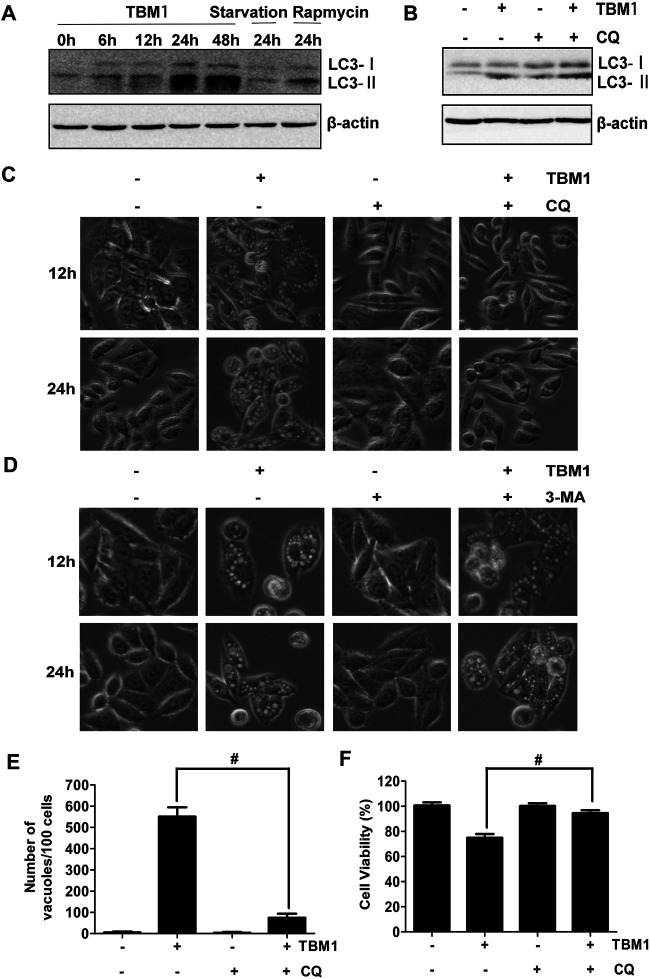
Chloroquine suppress TBM1-induced vacuolation and cell death in CRC cells. **(A)** SW480 were treated with 8 μM TBM1 for different time or starvation or 1 μM rapamycin for 24 h. Protein expression of LC3-I and LC3-II was detected by Western blotting. Beta-actin were served as loading control. **(B)** SW480 cells were pre-cultured with or without chloroquine (CQ) (10 μM) for 1 h before exposure to TBM1 for 24 h. LC3 processing was assessed using Western blotting analysis. **(C)** After treatment with SW480 cells for different time as **(B)**, morphological changes were observed by phase-contrast microscopy. **(D)** SW480 cells were pre-cultured with or without 3-MA (3 mM) for 1 h before exposure to TBM1 for different time. Morphological changes were observed using phase-contrast microscopy. **(E)** The number of vacuoles of the same cultures as **(B)** for 48 h was counted from three different imagines. **(F)** Cell viability was measured by MTT assay (*n* = 5). ^#^ *p* < 0.01.

The second image of bottom panel of Figure 6D was misused. The corrected Figure 6 appears below.

The authors apologize for this error and state that this does not change the scientific conclusions of the article in any way. The original article has been updated.

