# Supplementary material for: Tubeimoside 1 Acts as a Chemotherapeutic Synergist via Stimulating Macropinocytosis
Source: Front Pharmacol. 2018 Sep 26;9:1044. doi: 10.3389/fphar.2018.01044 (PMC6169148; doi:10.3389/fphar.2018.01044)
Supplement: Supplementary file 1 [file Table_1.docx]

**Supplementary material**

**1 Materials and Methods**

1.1 Cell lines and cell culture. Human colon carcinoma cell lines DLD-1 were purchased from the Chinese Academy of Science Committee Type Culture Collection Cell Bank (Shanghai, China). DLD-1 cells were cultured in RPMI 1640 medium (Gibco) supplemented with 10% fetal bovine serum (Gibco) and 1% penicillin-streptomycin (Invitrogen) at 37°C in a humidified incubator of 5% CO_2_-containing atmosphere.

1.2 Methods.

Liver function test. The mice were theated with TBM1 and/or 5-FU for 12h. The serum was collected and the serum alanine amino-transferase (ALT), aspartate amino-transferase (AST), alkaline phosphatase acid were detected by Guangzhou Kingmed Medical Test Center Co. Ltd.

Cell morphology and cell viability assay, Western blotting, uptake of Lucifer yellow and Organelle-specific Tracers, Time-lapse microscopy, are described in the Methods of the manuscript.

**2. Figures and legends**

Figure S1. TBM1 induces the formation of phase-lucent cytoplasmic vacuoles through macropinocytosis. **(A)** DLD-1 cells were treated with different concentrations of TBM1 for indicated time. Extensive accumulation of cytoplasmic vacuoles in DLD-1 cells were visualized using phase-contrast microscopy. **(B)** DLD-1 cells that treated with 8 μM TBM1 were monitored by time-lapse confocal microscope, two newly formed vacuoles marked with red or blue arrowheads fused into a larger vacuole, respectively. **(C)** DLD-1 cells were treated with 8 μM TBM1 for 24 h and then incubated with Lucifer yellow or organelle tracers. Lucifer yellow incorporated into phase-lucent vacuoles (left panel). ERtracker, lysotracker and mitotracker were used to label different subcellular organellae. The matching phase-contrast and fluorescent images were taken using inverted fluorescent microscope. **(D)** DLD-1 cells were treated with 8 μM TBM1 for 24 h and then incubated with dextran Af-488 for 4h. Cells were washed and cultured for 1.5 h with lysotracker Red. The phase-contrast image of the same field were consecutively monitored by microscope. **(E)** DLD-1 cells were pre-incubated in presence or absence of amiloride (1 mM) for 1 h before exposure to TBM1 for 24 h. The number of vacuoles was counted from three different microscopic fields.


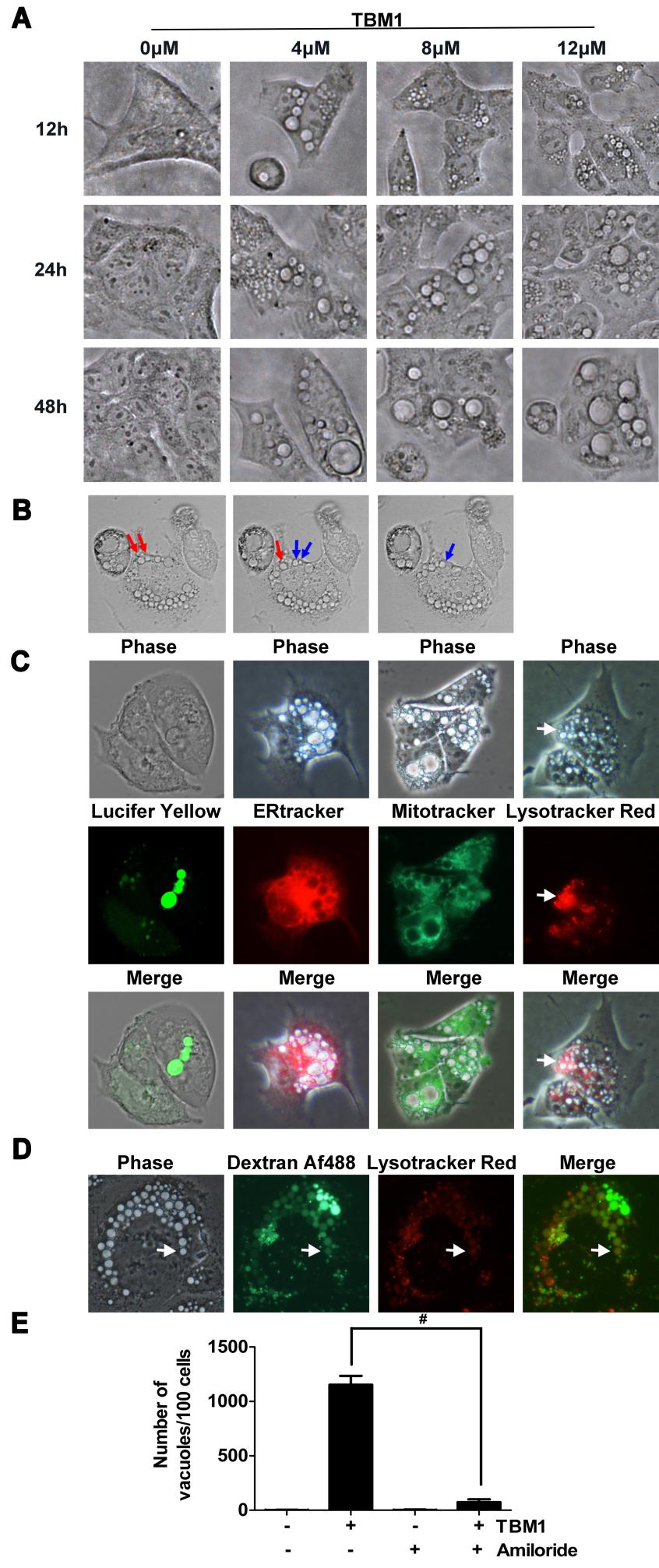


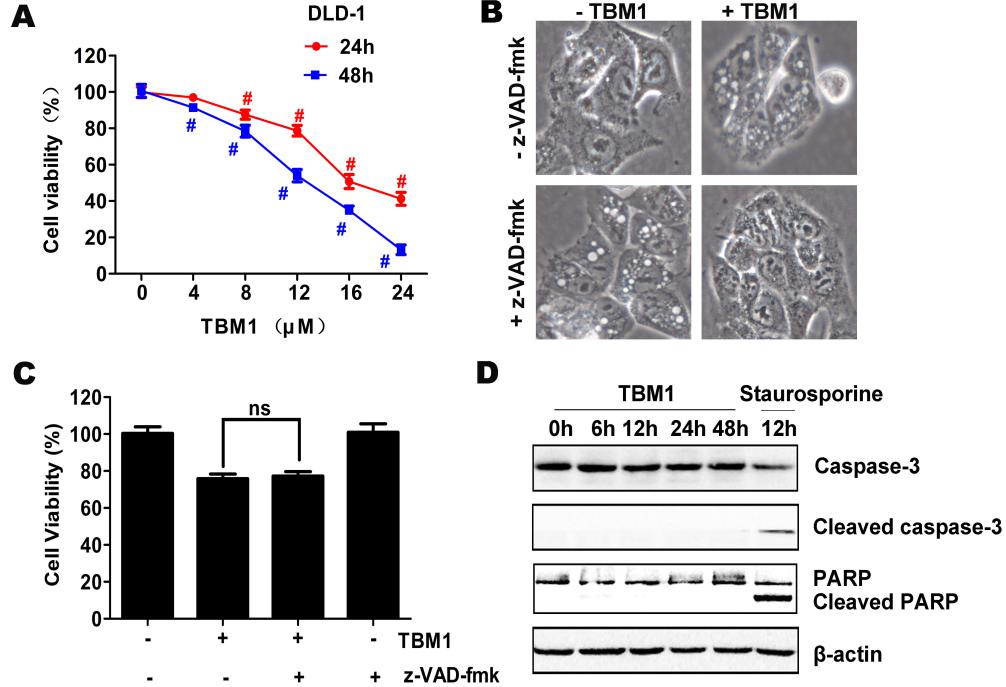


Figure S2. TBM1 induces caspase-independent cell death in DLD-1 cells. **(A)** DLD-1 cells were treated with different concentrations of TBM1 for 24 or 48 h. Cell viability was measured by MTT assay (*n* = 5). **(B-C)** DLD-1 cells were pre-cultured with or without pan-caspase inhibitor, z-VAD-fmk (50 μM) for 1 h before exposure to TBM1 for 48 h. Morphological changes were observed by phase-contrast microscopy (B). Cell viability was measured by MTT assay (*n* = 5)(C). **(D)** DLD-1 cells were treated with 8 μM TBM1 or 1 μM staurosporine for different time. Protein expression of caspase 3, cleaved caspase 3, PARP and cleaved PARP was detected by western blotting. β-actin were served as the loading control. Three independent experiments were performed and data presented were showed as means ± SD. ^#^, *p* < 0.01. ns shows that there is no significant differences.


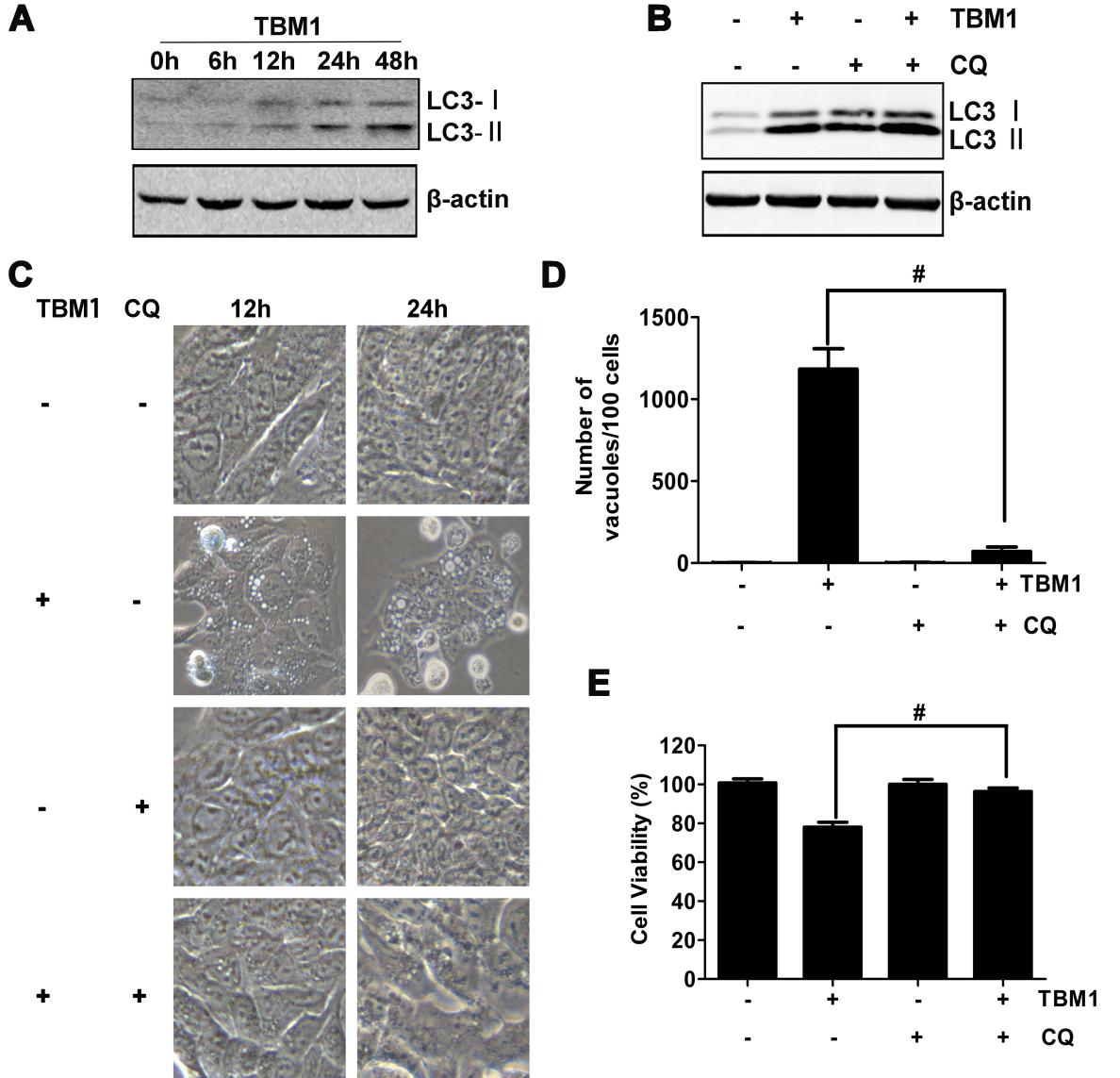


Figure S3. TBM1 recruits LC3-II to induce vacuolation in DLD-1 cells. **(A)** DLD-1 were dealed with 8 μM TBM1 from 0 to 48 h, starvation 24h, 1 μM rapamycin 24 h, respectively. Protein expression of LC3-I and LC3-II was detected by western blotting. Beta-actin were served as loading control. **(B-E)** DLD-1 cells were pre-incubated with or without CQ (10 μM) for 1 h before exposure to TBM1 for 24 h. LC3 processing was assessed using western blotting analysis (B). Morphological changes were observed by phase-contrast microscopy (C). The number of vacuoles was counted from three microscopic fields (D). Cell viability was measured by MTT assay (n = 5) (E) .


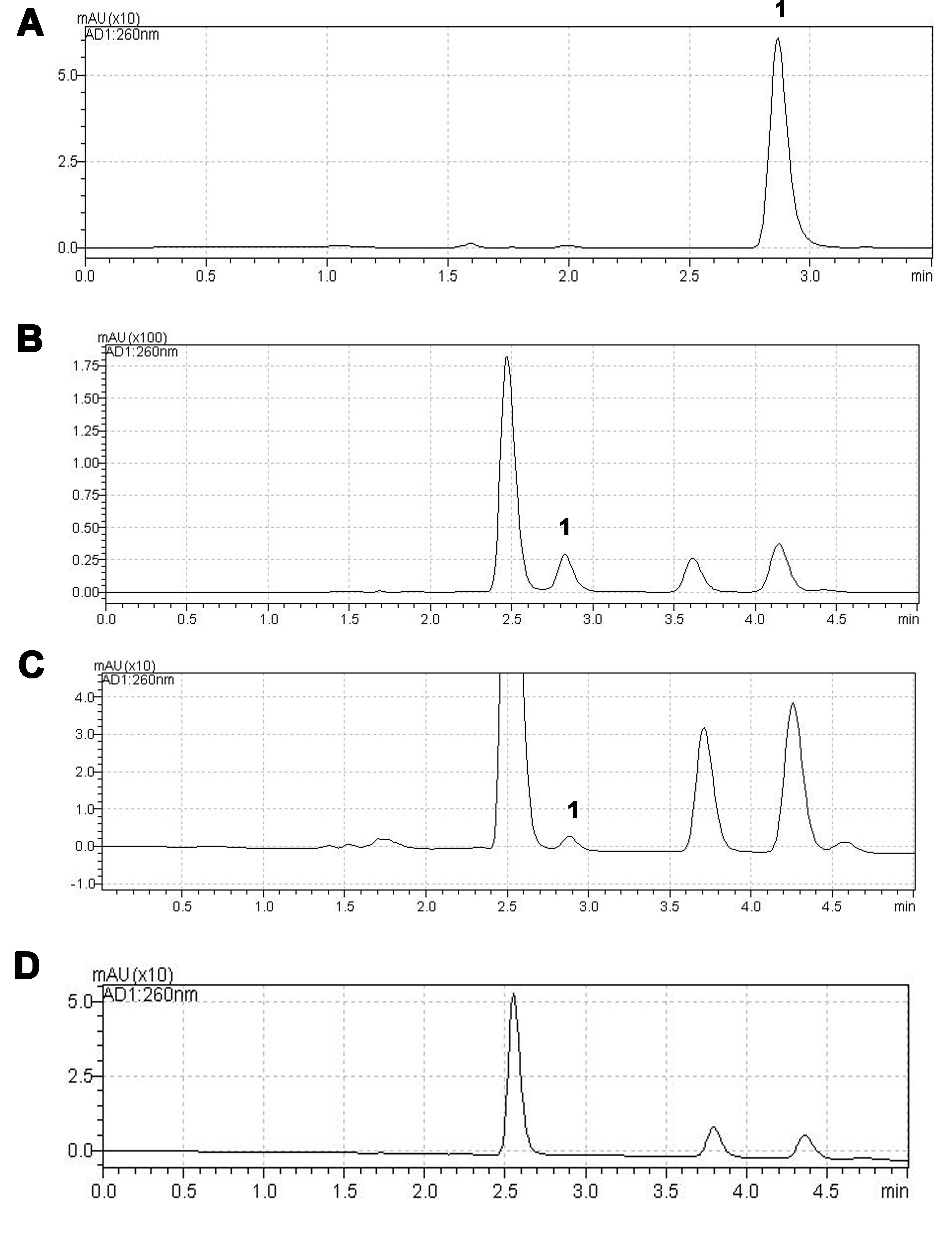


Figure S4. Chromatography of 5-Fu in cancer tissue by HPLC. **(A)** 5-Fu standard. **(B)** Cancer tissue spiked with 5-Fu. **(C)** Cancer tissue from mice by [intraperitoneal injection](#keyfrom=dict.basic.wordgroup) with the combination of 5 mg·kg^-1^ TBM1 and 5 mg·kg^-1^ 5-Fu. **(D)** Blank cancer tissue. Peak 1: 5-Fu.

Table S1 The hepatic function in mice treated with 5-FU and/or TBM1

| Groups | ALT(U·L^-1^) | AST(U·L^-1^) | ALP(U·L^-1^) |
| --- | --- | --- | --- |
| Control | 33.0±2.6 | 94.6±16.3 | 192.3±7.8 |
| TBM1 (2.5) | 33.0±10.7 | 77.7±18.5 | 156.7±14.6# |
| 5-Fu (2.5) | 25.0±10.0 | 79.7±6.8 | 184.3±13.9 |
| TBM1 (2.5) + 5-Fu (2.5) | 32.3±8.6 | 82.0±9.5 | 178.0±7.5 |
| TBM1 (5) | 31.0±8.5 | 86.7±23.4 | 162.3±16.3# |
| 5-Fu (5) | 28.3±7.8 | 76.7±5.7 | 182.3±12.2 |
| TBM1 (5) + 5-Fu (5) | 32.3±4.1 | 83.7±4.0 | 172.3±11.5 |
| TBM1 (2.5) + 5-Fu (5) | 35.3±6.1 | 70.7±18.1 | 172.7±10.6 |
| TBM1 (5) + 5-Fu (2.5) | 33.0±10.4 | 88.7±16.5 | 177.7±10.5 |
